# A Trypsin Family Protein Gene Controls Tillering and Leaf Shape in Barley^1^

**DOI:** 10.1104/pp.19.00717

**Published:** 2019-08-19

**Authors:** Lingzhen Ye, Yin Wang, Lizhi Long, Hao Luo, Qiufang Shen, Sue Broughton, Dianxing Wu, Xiaoli Shu, Fei Dai, Chengdao Li, Guoping Zhang

**Affiliations:** a Institute of Crop Science, Zhejiang University, Hangzhou 310058, China; b New Rural Development Institute, Zhejiang University, Hangzhou 310058, China; c Institute of Rural Development, Zhejiang Academy of Agricultural Sciences, Hangzhou 310021, China; d Key Laboratory of the Ministry of Agriculture for Creative Agriculture, Zhejiang Academy of Agriculture Sciences, Hangzhou 310021, China; e Western Barley Genetics Alliance/State Agricultural Biotechnology Centre, Murdoch University, Murdoch Western Australia 6132, Australia; f Department of Primary Industry and Regional Development, Government of Western Australia, Perth, Western Australia 6151, Australia; g State Key Laboratory of Rice Biology and Key Laboratory of the Ministry of Agriculture for Nuclear Agricultural Sciences, Zhejiang University, Hangzhou 310058, China; h Hubei Collaborative Innovation Centre for Grain Industry, Yangtze University, Hubei Jingzhou 434025, China

## Abstract

Tillering or branching is an important agronomic trait in plants, especially cereal crops. Previously, in barley (*Hordeum vulgare*) ‘Vlamingh’, we identified the *high number of tillers1* (*hnt1*) mutant from a γ-ray-treated segregating population. *hnt1* exhibited more tillers per plant, narrower leaves, and reduced plant height compared with the wild-type parent. In this study, we show that the *hnt1-*increased tiller number per plant is caused by accelerated outgrowth of tiller buds and that *hnt1* narrower leaves are caused by a reduction in vascular tissue and cell number. Genetic analysis revealed that a 2-bp deletion in the gene *HORVU2Hr1G098820* (*HvHNT1*), encoding a trypsin family protein, was responsible for the *hnt1* mutant phenotype. Gene function was further confirmed by transgenic complementation with *HvHNT1* and RNA interference experiments. *HvHNT1* was expressed in vascular tissue, leaf axils, and adventitious root primordia and shown to negatively regulate tiller development. Mutation of *HvHNT1* led to the accumulation of a putative cyclophilin-type peptidyl-prolyl cis/trans-isomerase (HvPPIase), which physically interacts with the HvHNT1 protein in the nucleus of plant cells. Our data suggest that *HvHNT1* controls tiller development and leaf width through *HvPPIase*, thus contributing to understanding of the molecular players that control tillering in barley.

## Abstract (precis)

A mutant screen for high tiller number in barley isolates a gene controlling tiller development.

Tillering or branching is an important agronomic trait that heavily influences crop yield. It is well documented that the yield of cereal crops, including wheat (*Triticum aestivum*; Abichou et al., 2018), rice (*Oryza sativa*; Clerget et al., 2016), and barley (*Hordeum vulgare*; Sadras and Slafer, 2012), can be improved by manipulating tillering. The Green Revolution, characterized by the development of semidwarf rice and wheat cultivars, dramatically increased yield potentials by enhancing tillering and thus the formation of spikes (Reynolds et al., 2011; Pingali, 2012). Tillers in grass plants are initiated and developed from the axillary meristem of basal nodes with short-elongated internodes (Grbić and Bleecker, 2000). Each tiller has the potential to grow independently of the primary stem, by virtue of its adventitious roots, to generate its own spike (Kebrom et al., 2012). Moreover, tillering is also important for cereal crop plants to enhance their competitiveness with weeds.

There are two stages in tiller development, bud formation and outgrowth. In recent years, analyses of mutants from various plant species have revealed that many genes are involved in the regulation of each stage. Mutants such as *barren stalk1* (*ba1*; Gallavotti et al., 2004) and *barren inflorescence2* (*bif2*; McSteen et al., 2007) in maize (*Zea mays*) and *monoculm1* (*moc1*; Li et al., 2003) in rice lost their ability to form tiller buds due to intrinsic defects in axillary meristem initiation, indicating that bud formation is genetically controlled. In contrast, axillary bud outgrowth is regulated by genes, phytohormones, environmental factors, and their interactions. Several genes that inhibit the outgrowth of tiller buds have been identified in some mutants, including *Teosinte branched1* (*Tb1*; Doebley et al., 2006) and *Grassy tillers1* (*gt1*; Whipple et al., 2011) in maize and *Teosinte Branched1* (*OsTB1*; Takeda et al., 2003), *Ideal Plant Architecture1* (*ipa1*; Jiao et al., 2010; Miura et al., 2010), *Dwarf27* (*D27*; Lin et al., 2009), *D14*/*HTD2* (Arite et al., 2009), and *D53* (Jiang et al., 2013; Zhou et al., 2013) in rice. The *TB1*-like genes inhibit bud outgrowth in response to several dormancy-inducing hormonal and environmental signals, indicating their role as an integrator of signals controlling dormancy versus the outgrowth fate of an axillary bud (Takeda et al., 2003). Rice *dwarf* (*d14*, *d27*, and *d53*) mutants had significantly more tillers than their wild-type counterparts, which was closely associated with phytohormone metabolism, including strigolactone (Jiang et al., 2013; Zhou et al., 2013). Moreover, the wheat *tiller inhibition* (*tin*) mutant was characterized by precocious internode elongation that diverts Suc from the developing tillers (Kebrom et al., 2012). The *tin* gene has no direct effect on the initiation of axillary meristems or bud outgrowth but instead regulates tillering indirectly by controlling the timing of internode elongation. In short, tiller development is regulated by many factors, and continued identification of mutants affected in tillering may reveal further genes controlling tiller development.

Barley is the fourth largest cereal crop in terms of planting area in the world. It has been used as a Triticeae model plant because of its diploid genome. Compared with other Triticeae species, such as wheat and rye (*Secale cereale*), barley has a low tillering capacity and fewer spikes per plant. Although several tiller mutants have been identified, only a few relevant genes, such as *UNICULME4* (*Cul4*) that reduces tiller number per plant (Tavakol et al., 2015) and *ELIGULUM-A* (*ELI-A*) that regulates leaf and tiller development (Okagaki et al., 2018), have been identified. In addition, the candidate gene *JuBel2* was identified in the *low number of tillers1.a* mutant, which encodes a BELL-like homeodomain transcription factor (Dabbert et al., 2010). Moreover, the homologous gene of *TB1* in barley (named *INT-C*) has a dramatic influence on lateral spikelet fertility but a minor effect on tiller development (Ramsay et al., 2011). Therefore, more tiller-related mutants and gene isolation in barley will enrich the understanding of barley tiller development.

In a previous study, we identified a high-tillering mutant of barley ‘Vlamingh’ isolated from a segregating population treated with γ-rays. In addition to more tillers per plant, this mutant, designated *high number of tillers1* (*hnt1*), also had narrower leaves and shorter plants than the wild-type parent. In this study, we investigated the genetics behind the *hnt1* mutant phenotype. We first identified the candidate gene of *HvHNT1* on chromosome 2HL by molecular mapping, then confirmed that *HvHNT1* encodes a trypsin family protein using transgenic analysis. The expression pattern of *HvHNT1* was highly associated with tiller formation and leaf development. We identified a putative cyclophilin-type peptidyl-prolyl cis/trans-isomerase (HvPPIase), which is considered the substrate of the HvHNT1 protein. Accordingly, we assume that *HvHNT1* controls tiller development and leaf width in barley through regulating HvPPIase.

## RESULTS

### Phenotyping of the *hnt1* Mutant

**Figure 1.**
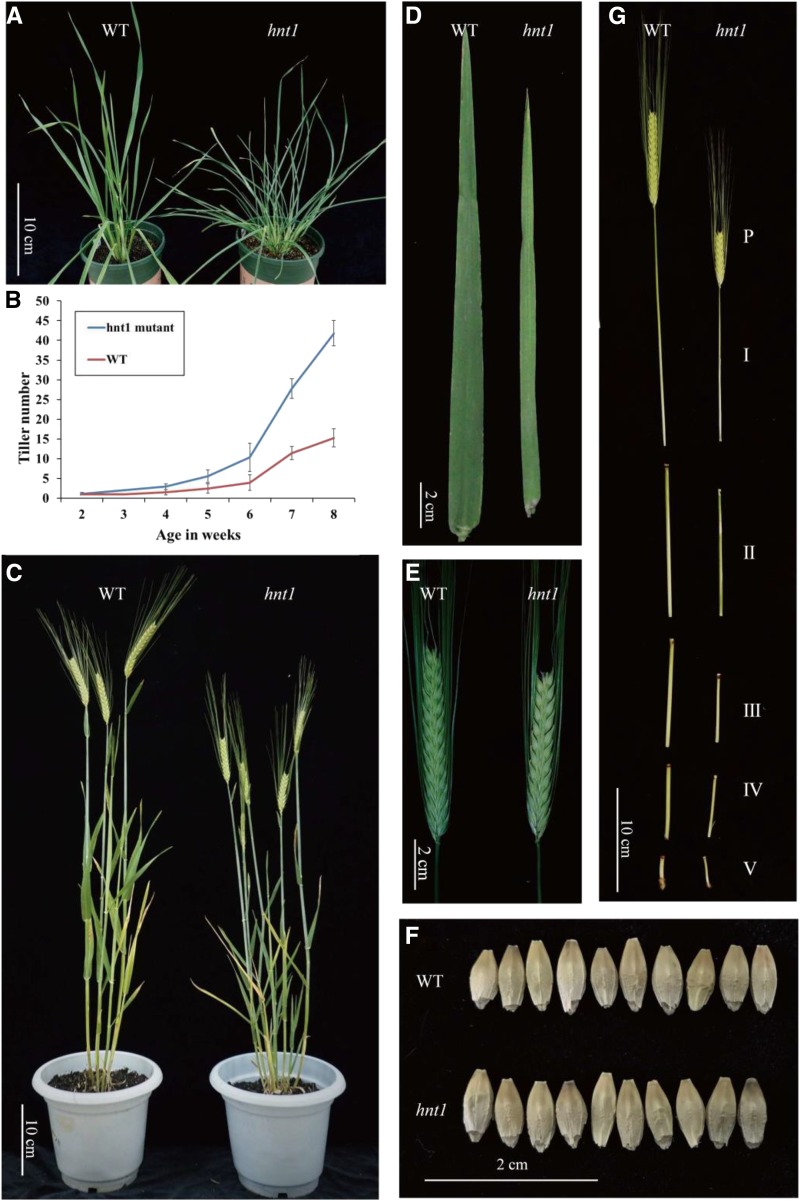
Phenotypes of the *hnt1* mutant and its wild-type parent. A, Gross morphology at the tillering stage. B, Total tiller number of *hnt1* and wild-type (WT) plants from 2 to 8 weeks after sowing. Values shown are means ± sd with 12 biological replicates. C, Gross morphology at the heading stage. D, Leaf. E, Spike. F, Seed. G, Internodes. Bars = 10 cm (A, C, and G) and 2 cm (D–F).

The *hnt1* mutant exhibited more tillers per plant, narrower leaves, and shorter plants than the wild type (Fig. 1; Table 1). The *hnt1* mutant had several fold more tillers per plant than the wild type at all sampling times (Fig. 1B). Leaf length showed no significant difference between the *hnt1* mutant and the wild type; however, leaf width of the mutant was approximately half that of the wild type (Fig. 1D). The *hnt1* mutant plants had shorter internode lengths than the wild type, hence the shorter plants (Fig. 1G; Table 1). Moreover, the *hnt1* mutant had lower grain weights than the wild type (Fig. 1F; Table 1).

**Table 1.** Morphological differences between *hnt1* mutant and wild-type plants The comparison was undertaken at the heading stage for all traits, except total tiller number and 1,000 kernel weight, which were measured at the stem elongation stage and maturity, respectively. Data are presented as means ± sd using 12 biological replicates. Asterisks indicate significant differences between wild-type and *hnt1* plants as determined by Student’s *t* test: **, *P* < 0.01.

Trait	*hnt1*	Wild Type
Plant height (cm)	49.24 ± 2.46	64.76 ± 4.32**
Leaf width (cm)	0.52 ± 0.03	0.93 ± 0.08**
Leaf length (cm)	11.66 ± 1.11	12.61 ± 1.02
Internode I length (cm)	15.04 ± 0.63	22.53 ± 0.9**
Internode II length (cm)	12.07 ± 0.39	14.33 ± 0.46**
Internode III length (cm)	6.84 ± 0.68	10.37 ± 0.71**
Internode IV length (cm)	5.68 ± 0.74	7.25 ± 0.51**
Internode V length (cm)	2.84 ± 0.25	3.09 ± 0.13
No. of fertile tillers	9.40 ± 1.03	5.12 ± 0.89**
Total tiller number	40.35 ± 4.61	15.68 ± 3.43**
1,000 kernel weight (g)	34.43 ± 1.13	38.18 ± 1.49**

**Figure 2.**
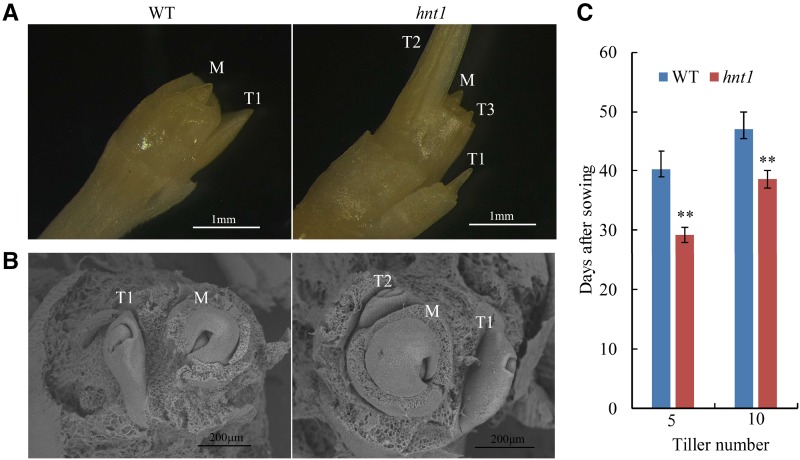
Tiller development in the *hnt1* mutant and its wild-type parent. A, Morphology of *hnt1* and wild-type (WT) stem bases containing tiller buds. B, Scanning electron microscopy images of the stem base. M, Main stem; SAM, shoot apical meristem; T1, first tiller; T2, second tiller; T3, third tiller. C, Number of days to reach five and 10 tillers.

To elucidate the mechanisms behind high tillering in the *hnt1* mutant, the base tissues including tiller buds were examined histologically. In 15-d-old seedlings, the *hnt1* mutant had two or more tillers per plant, with one tiller bud in each leaf axil, whereas the wild type had only one small tiller bud (Fig. 2A). Similarly, the *hnt1* mutant had more tiller bud primordia than the wild type when examined with a scanning electron microscope (Fig. 2B). A quantitative analysis of bud and tiller development showed that the *hnt1* mutant took significantly fewer days to produce the same number of tillers than the wild type (Figs. 1B and 2C), indicating that tiller development in the *hnt1* mutant is significantly faster than in the wild-type plants. Since both the *hnt1* mutant and the wild type had only one tiller bud in each leaf axil, more tillers per plant in the *hnt1* mutant can be attributed to more rapid outgrowth of tiller buds (Figs. 1B and 2).

**Figure 3.**
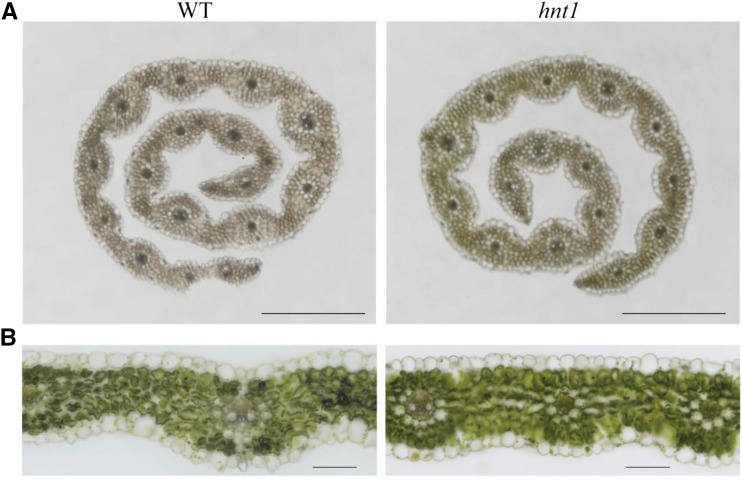
Leaf anatomy of the *hnt1* mutant and its wild-type parent. Transverse sections are shown for rolled (A) and mature (B) *hnt1* and wild-type (WT) leaves. The number of veins in a leaf was counted in rolled and mature leaves. The epidermal cell number between two adjacent veins was counted in mature leaves. Bars = 500 μm (A) and 100 μm (B).

Anatomical observations found significantly fewer veins per leaf on average for the *hnt1* mutant (14.8) than for the wild type (17.8; *P* < 0.01; Fig. 3A). Furthermore, the mutant had 11.2 epidermal cells between two adjacent veins, significantly fewer than those of the wild type (14.4; *P* < 0.01; Fig. 3B). However, no significant difference between *hnt1* and the wild type was observed regarding epidermal cell size. Thus, the narrower leaves of the *hnt1* mutant are attributed to fewer veins and cells, indicating that the mutant is affected in cell division rather than cell elongation.

### Mapping and Identification of *HvHNT1*

To map the *hnt1* locus, a double haploid (DH) mapping population was developed from a cross of the *hnt1* mutant and cv Boluke. The segregation ratio of mutant and normal plants, specifically 148 of the 269 DH lines exhibiting the mutant phenotype, suggested that *hnt1* is controlled genetically by a single locus. In addition, the plants with more tillers were consistently associated with narrower leaves and shorter stature.

**Figure 4.**
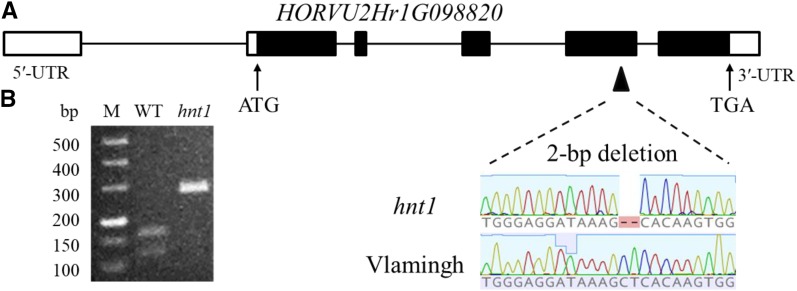
Diagram of the *HORVU2Hr1G098820* gene in the *hnt1* mutant and its wild-type parent. A, Sequence differences between the wild type (WT) and the *hnt1* mutant. Black boxes represent gene exons. UTR, Untranslated region. B, CAPS marker differentiating the wild type and the *hnt1* mutant. M, DNA ladder.

Linkage analysis using 360 molecular markers showed that the *hnt1* locus was primarily located in an interval between microsatellite marker Ebmac0039 and insertion/deletion marker M458438 on the long arm of chromosome 2H. To narrow the locus region, 25 new markers were developed, and the *hnt1* locus was located in a region between insertion/deletion markers M39589 and M54556 (Supplemental Table S1). Within this region, there are 32 high-confidence genes (Supplemental Table S2) according to the results reported by Mayer et al. (2012) and (Mascher et al., 2017). A 2-bp deletion in a putative gene, *HORVU2Hr1G098820* (*MLOC_67307*), was detected after cloning and sequencing the candidate genes in the *hnt1* mutant and the wild type (Fig. 4A). The 2-bp deletion at the fourth exon caused a frameshift and generated a premature translation termination product in the *hnt1* mutant allele. A cleaved-amplified polymorphic sequence (CAPS) molecular marker was developed to screen the genotypes with the 2-bp deletion. As a result, the PCR product of the wild type was 267 bp and could be digested into 153-bp and 114-bp fragments by endonuclease *Alu*I. In contrast, the *Alu*I-digested product of the *hnt1* mutant was 265 bp, as the 2-bp deletion disrupted the *Alu*I restriction enzyme cutting site (Fig. 4B). All lines of the DH population were screened with the CAPS marker, which cosegregated with the phenotype. Therefore, *HORVU2Hr1G098820* was considered the candidate gene responsible for the *hnt1* mutant phenotype, herein denoted *HvHNT1*.

### Gene Validation of *HvHNT1*

To confirm that the 2-bp deletion in *HvHNT1* was responsible for the *hnt1* mutant phenotype, a genetic complementation analysis was performed. A 6.75-kb genomic DNA fragment containing a promoter (3,078-bp upstream sequence), coding region (3,348 bp), and 3′ untranslated region (324-bp downstream sequence) of the *HvHNT1* gene was introduced into the *hnt1* mutant through *Agrobacterium tumefaciens*-mediated transformation.

**Figure 5.**
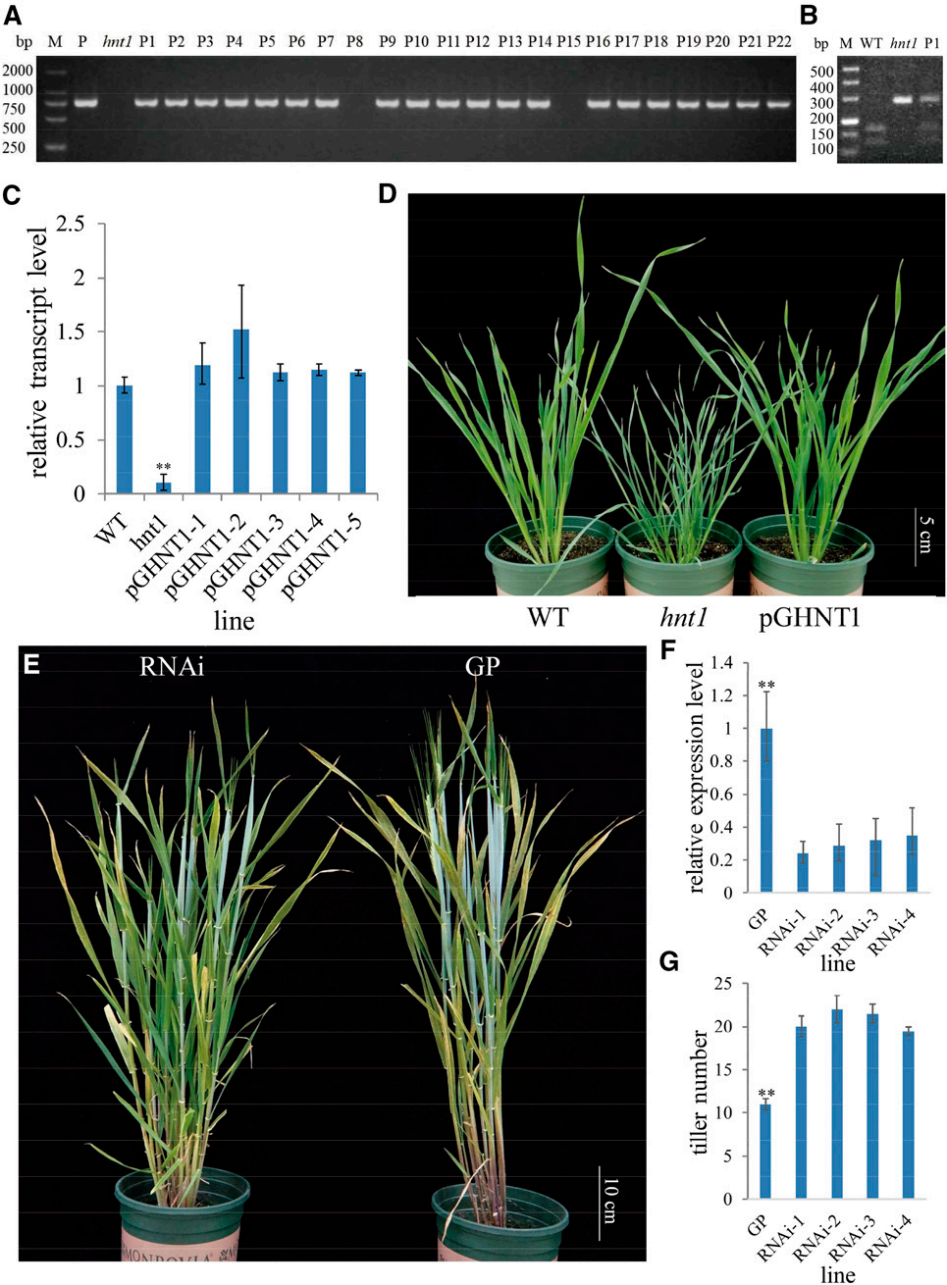
Transgenic analysis of the *hnt1* mutant. A to D, Genetically complementary transformation experiments. A, Genomic PCR amplification of the transgenic seedlings. *hnt1*, Mutant, negative control; M, DNA ladder; P, positive control, constructed complementary vector; P1 to P22, 22 lines of T0 plants. B, CAPS marker in the wild type (WT; cv Vlamingh), *hnt1* mutant, and positive transgenic line. C, Relative gene expression levels in leaves of the wild type, *hnt1* mutant, and positive T1 lines (pGHNT1–pGHNT5) by RT-qPCR analysis. The difference between the *hnt1* mutant and other seedlings is significant (*P* < 0.01) by Student’s *t* test. D, Phenotypes of the wild type, *hnt1* mutant, and positive T1 lines (pGHNT1) at tillering. E to G, Information on the *HvHNT1*-RNAi lines and its wild-type counterpart (cv Golden Promise [GP]). E, Phenotypes of RNAi lines and GP at the heading stage. F, Relative gene expression levels in the leaves of GP and the positive RNAi lines by RT-qPCR analysis. G, Tiller number of GP and positive RNAi lines. Significant differences between RNAi lines and GP are shown: **, *P* < 0.01 by Student’s *t* test.

The sequence information of the constructed genetic complementation vector was used to develop a PCR-based marker, Re_F/R (Supplemental Table S1), for determining the genetic background in the complementation test. Of the 22 transgenic plants, 20 lines were confirmed by genomic PCR as positive transgenic seedlings (Fig. 5A). The identified 20 primary lines (T0) exhibited phenotypes similar to the wild type, thus demonstrating complementation of *hnt1*. The CAPS marker was also used to verify the transformed lines; the positive T0 seedlings were the heterozygote of the mutant and wild-type alleles of *HvHNT1* (Fig. 5B). Five of these lines were selected to generate T2 seedlings for further characterization. Reverse transcription quantitative PCR (RT-qPCR) analysis of the *HvHNT1* gene showed that the complemented lines had much higher expression levels than the mutant and no significant difference from the wild type (Fig. 5C). In addition, the complemented phenotype was heritable in the T1 plants (Fig. 5D), thus supporting that the *hnt1* mutant phenotype is caused by mutation of the *HvHNT1* gene.

To further examine the role of *HvHNT1*, we used an RNA interference (RNAi) approach to inhibit *HvHNT1* expression. Several *HvHNT1*-RNAi lines were obtained, and the corresponding wild type (cv Golden Promise) was used as the control for each one. Four independent and positive T2 lines were selected for further characterization. The expression level of *HvHNT1* in these four RNAi lines ranged from 24% to 34.9% of that in the wild type (Fig. 5F), and tillers per plant ranged from 19.5 to 22, being significantly higher than in the wild type (11 tillers per plant; Fig. 5G). Meanwhile, leaf width and plant height did not significantly differ between the RNAi lines and the wild type (Fig. 5E). These findings indicate that the phenotypic change in *HvHNT1* knockdown lines is in partial consensus with the *HvHNT1* knockout line (*hnt1* mutant) and that *HvHNT1* negatively regulates tiller development in barley.

### *HvHNT1* Encodes a Trypsin Family Protein

The sequence comparison between genomic DNA and cDNA revealed that *HvHNT1* comprised five exons and four introns (Fig. 4A). The gene was predicted to encode a trypsin family protein with 648 amino acids, and bioinformatic analysis showed that the amino acid sequence 210 to 425 was the functional domain region using the InterPro online search tool (https://www.ebi.ac.uk/interpro/).

**Figure 6.**
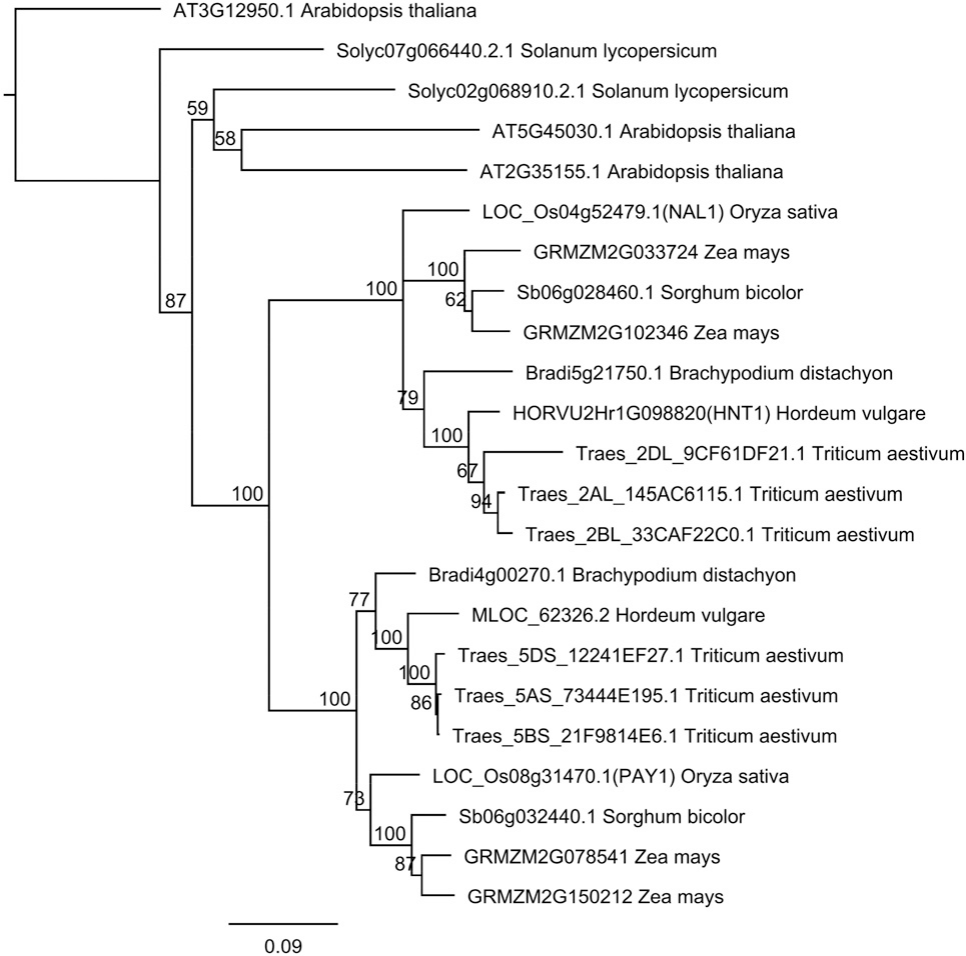
Phylogeny of the HNT1 protein family. A neighbor-joining tree was built using amino acid sequences of predicted proteins from Arabidopsis, tomato, maize, sorghum, wheat, *B. distachyon*, barley, and rice using GENEIOUS 10.0.

A BLAST analysis was performed on the HNT1 protein sequence of different plant species. Based on the predicted protein sequences, a phylogenetic tree was constructed to reveal the evolutionary relationship of the gene family members (Fig. 6). Another homologous gene became apparent in the barley genome, namely *MLOC_62326.2*, which shared 69.4% similarity to *HvHNT1* in the amino acid sequence. Moreover, the *HvHNT1* gene in barley shared high sequence similarity with that in other species of Gramineae, including wheat, maize, rice, sorghum (*Sorghum bicolor*), and *Brachypodium distachyon*. In wheat, each subgenome has two homologous genes, where the genes from chromosome 2 in each subgenome share the closest genetic relationship. In addition, homologous proteins of HvHNT1 were also found in other plants, including Arabidopsis (*Arabidopsis thaliana*) and tomato (*Solanum lycopersicum*). The rice *narrow leaf1* (*nal1*) mutant with narrow-leaf and dwarf phenotype (Qi et al., 2008) showed a degree of phenotypic similarity to the *hnt1* mutant. The existence of *HNT1*-like genes in other plant species and the partial phenotypic similarity of the *nal1* rice mutant and *hnt1* barley mutant indicate partial conservation of biological functions for the HvHNT1 protein family.

### Expression Pattern of *HvHNT1*

**Figure 7.**
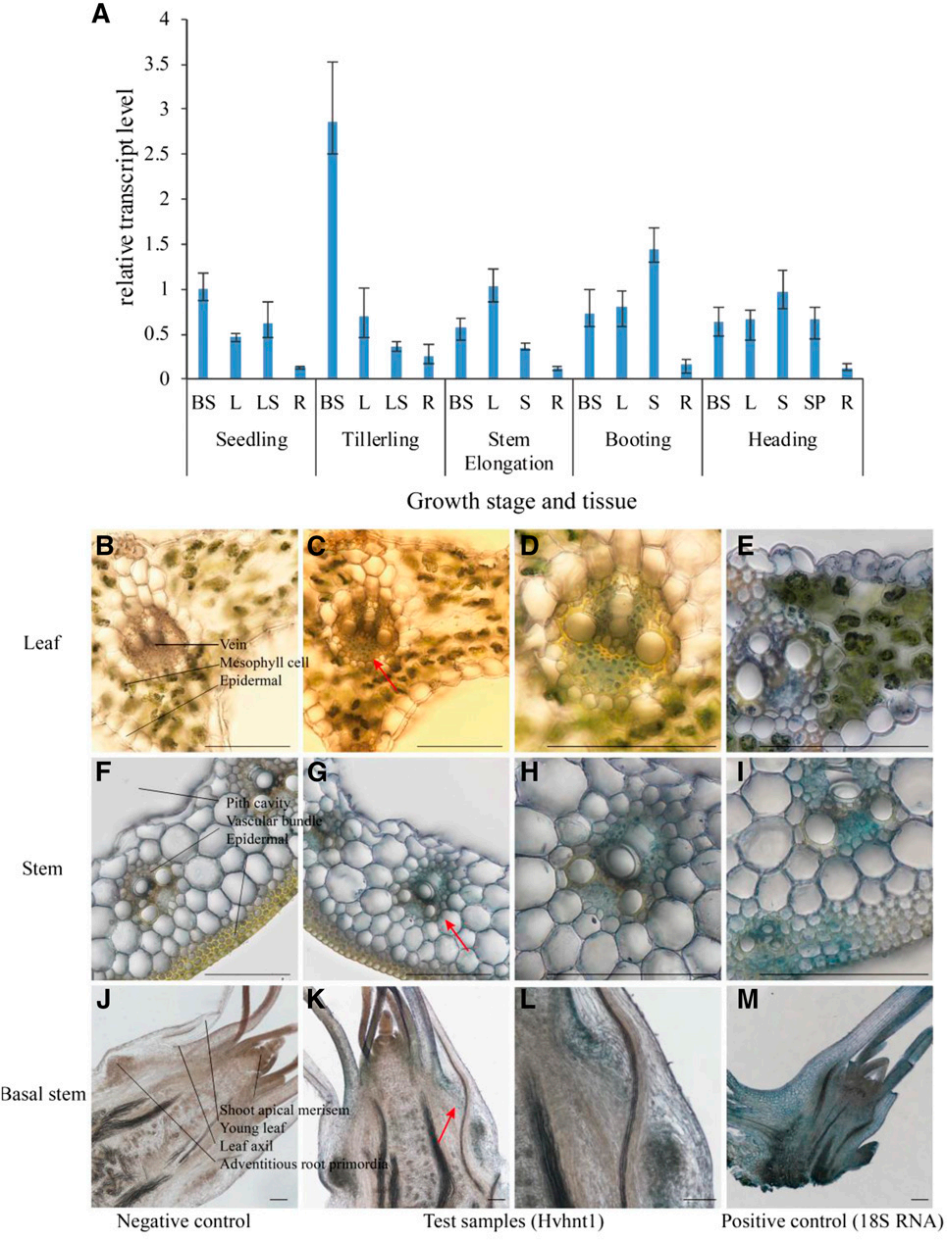
*HvHNT1* expression in various tissues at different growth stages. A, Total RNAs isolated from the basal stem (BS), leaf (L), leaf sheath (LS), stem (S), spike (SP), and root (R). RT-qPCR analysis measured the relative mRNA levels, which were normalized to the transcript levels of *HvACTIN*. Mean and sd values were obtained from three biological replicates. The experiment was repeated twice. B to M, In situ PCR of *HvHNT1* in different tissues of wild-type barley ‘Vlamingh’. All samples were stained with BM Purple. The blue/purple color indicates the presence of digoxigenin (DIG)-labeled cDNA, and brown indicates the absence of the amplified cDNA target. Images C, D, G, H, K, and L are test samples. The right lane images are enlarged visions of the left lane, especially the red arrows indicating area (signal area, *HvHNT1* expression sites). Images B, F, and J are negative controls where RT was omitted. Images E, I, and M are positive controls with 18S RNA to show staining in all cells. Images B to E are cross sections of leaves at the seedling stage, showing the detection of *HvHNT1* mainly in the vascular system (veins). Images F to I are cross sections of stems at the booting stage, showing the detection of *HvHNT1* mainly in the vascular bundle of the stem. Images J to M are longitudinal sections of basal stems at the seedling stage, showing the detection of *HvHNT1* mainly in the leaf axil and adventitious root primordia. Bars = 100 μm.

To examine the expression pattern of the *HvHNT1* gene, total RNA was isolated from the basal stem, leaf, leaf sheath, root, stem, and spike of wild-type plants at different growth stages, including seedling, tillering, stem elongation, booting, and heading. Tillers grow from the nodes of unelongated internodes in the base stem near the shoot apical meristem, axillary meristem, and tiller buds. The results of RT-qPCR analysis showed that *HvHNT1* was mainly expressed in shoots, with low expression in roots at the seedling and tillering stages (Fig. 7A). At the heading stage, there was no obvious difference in the gene expression levels between the base stem and other tissues. In short, the *HvHNT1* gene was mainly expressed in the base stem at the tillering stage. As tillers are formed on the basal nodes, the high expression level of *HvHNT1* in seedling bases indicates that the gene is involved in tillering.

Based on the RT-qPCR results, cellular localization of *HvHNT1* was further determined using different tissues of barley ‘Vlamingh’, including leaf, stem, and basal stem, by in situ PCR analysis (Fig. 7, B–M; Supplemental Fig. S1). The presence of the transcripts was visualized by the color reaction of the alkaline phosphates bound to the antibody, which is specific to the DIG group incorporated during the amplification of the PCR product from specific cDNA produced by the specific reverse primers (Q-HNT1-F2/R2) that recognize *HvHNT1*. Cross sections of leaves and stem revealed that *HvHNT1* was mainly expressed in the vascular system. A longitudinal section of the basal stem at the seedling stage showed that *HvHNT1* was mainly expressed in the leaf axil and adventitious root primordia and thus highly related to vascular, tiller, and adventitious root formation. Therefore, *HvHNT1* expression is highly associated with tiller development and leaf width.

### HvHNT1 Might Regulate Barley Tiller Development through HvPPIase

**Figure 8.**
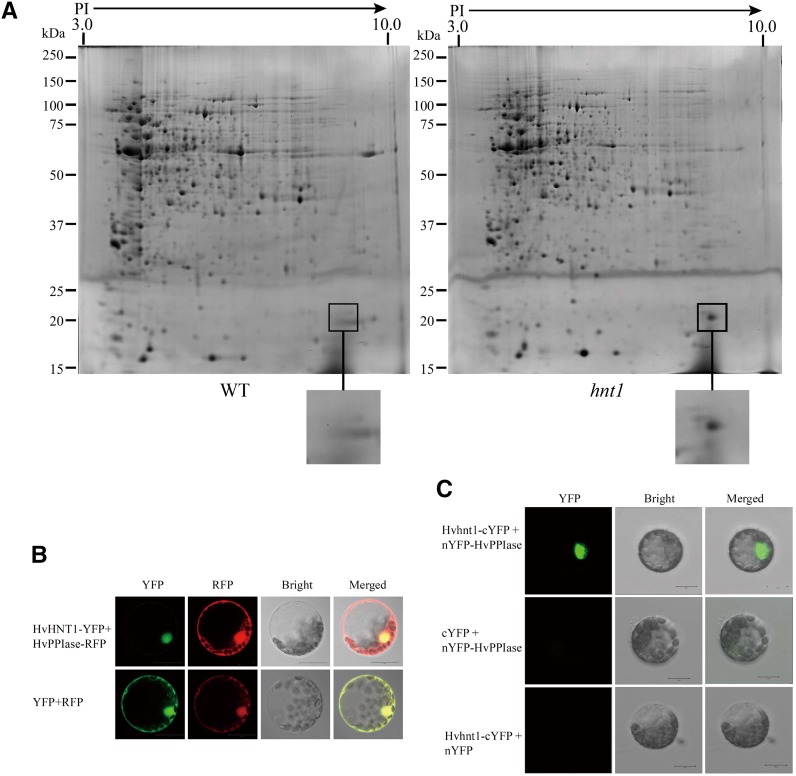
Interaction between HvHNT1 and HvPPIase. A, Two-dimensional electrophoresis of total proteins from the basal stems of four-leaf-aged wild-type (WT) and *hnt1* mutant seedlings. The frame area indicates the significant difference between the *hnt1* mutant and the wild type; the protein spot in *hnt1* was cut for LC-MS analysis. B, Subcellular localization patterns of HvHNT1 and HvPPIase in barley leaf protoplasts. Transient coexpression of HvHNT1-YFP and HvPPIase-RFP in protoplasts suggests that the two different fusion proteins are overlapping in subcellular localization. C, BiFC assays showed that HvHNT1 interacts with HvPPIase in barley protoplasts. Coexpression of nYFP-HvPPIase and cYFP, nYFP, and HvHNT1-cYFP was used as a negative control. Bars = 20 μm.

To understand the possible pathway involved in the high-tillering *hnt1* mutant phenotype, proteomic analysis was conducted using the basal stems of four-leaf-aged wild-type and *hnt1* mutant plants. The results showed that the wild type and the *hnt1* mutant had similar protein profiles except for an 18-kD protein (Fig. 8A), which was more abundant in the *hnt1* mutant. The protein spot was excised and digested by trypsin for further analysis using liquid chromatography-mass spectrometer (LC-MS). The accumulated protein in the mutant was identified as a putative cyclophilin-type HvPPIase. However, the RT-qPCR analysis showed that the transcriptional level of *HvPPIase* did not differ significantly between *hnt1* and the wild type. It may be assumed that HvPPIase is negatively regulated by HvHNT1 at the posttranscriptional level. The homologous gene of *HvPPIase* is *DIAGEOTROPICA* (*DGT*) in tomato. Mutation of the *DGT* gene resulted in pleiotropic phenotypes, including apical dominance reduction and vascular development alteration (Balbi and Lomax, 2003; Lavy et al., 2012). Therefore, it is logical that the accumulation of HvPPIase protein could increase tiller number and alter the leaf pattern in the *hnt1* mutant.

We further determined the subcellular localization pattern of HvHNT1 and HvPPIase by examining transient coexpression of HvHNT1-YFP and HvPPIase-RFP in barley leaf protoplasts and observed their localization using confocal microscopy. The fluorescence signal of HvHNT1-YFP was detected in the nucleus, whereas the fluorescence signal of HvPPIase-RFP was detected in the cytomembrane, cytoplasm, and nucleus (Fig. 8B). The overlay of HvHNT1-YFP and HvPPIase-RFP fluorescence signals in the cell nucleus suggests that the HvHNT1 and HvPPIase proteins are colocalized and may physically interact with each other in the nucleus (Fig. 8B).

To visualize the physical interaction of HvHNT1 and HvPPIase, a bimolecular fluorescence complementation (BiFC) assay was performed. For the BiFC assay, nonfluorescent N-terminal YFP fragment (nYFP) was fused to the C terminus of HvPPIase to create the nYFP-HvPPIase fusion, and the N terminus of HNT1 was fused to nonfluorescent C-terminal YFP fragment (cYFP) to create the HvHNT1-cYFP fusion. Interactions between these two fusion proteins were then transiently coexpressed in barley protoplasts. As a result, a strong YFP signal was detected using confocal microscopy (Fig. 8C). Coexpression between nYFP-HvPPIase and cYFP and between nYFP and HvHNT1-cYFP was used as a negative control. Therefore, the results of the BiFC assay suggest that HvHNT1 and HvPPIase physically interact in the nucleus of plant cells. Moreover, the in situ PCR analysis showed that the gene expression pattern of *HvPPIase* was partly similar to *HvHNT1*, with expression mainly in the vascular system and small tillers (Supplemental Fig. S2).

The HNT1 protein belongs to the trypsin protein family. The trypsin-like protease superfamily is defined by its cleavage of substrates with Arg or Lys as the site with broad-spectrum cleavage activity (Lin et al., 1999). The HvPPIase protein has 171 amino acids, including 14 Lys and six Arg. These amino acid sites are the potential cleavage sites for HNT1 protein. As HvHNT1 and HvPPIase interact physically in the nucleus and the HvPPIase protein accumulated in the *hnt1* mutant, we propose that HvPPIase is a substrate that may be digested by HvHNT1, thus underlying the mechanism through which *HvHNT1* regulates barley tiller development and leaf width.

## DISCUSSION

Several genes controlling tiller development in barley have been identified using low-tillering or even uniclum mutants (Tavakol et al., 2015; Okagaki et al., 2018). In this study, the *HvHNT1* gene was identified that appears to be closely related to high-tillering ability in barley. Compared with the wild type, the *hnt1* mutant has more tillers per plant and displays narrower leaves and shorter plant stature. Genetic analysis showed that the mutant phenotype is controlled by *HORVU2Hr1G098820*, which was denoted *HvHNT1* and encodes a trypsin family protein consisting of 648 amino acids. Transgenic analysis demonstrated that a 2-bp deletion in *HvHNT1* is responsible for the *hnt1* mutant phenotype. Moreover, the *HvHNT1* gene is highly expressed in the shoot base at the tillering stage and preferentially expressed in vascular tissues, leaf axils, and adventitious root primordia. Proteomic analysis, as well as BiFC and bioinformatics analyses, showed that HvPPIase physically interacts with and may be digested by HvHNT1. This was further validated by in situ PCR analysis of *HvPPIase* expression. Therefore, HvPPIase accumulated specifically in the *hnt1* mutant. In short, we propose that HvHNT1 affects barley tiller development through its regulation of HvPPIase.

In addition to controlling tiller formation, the *HvHNT1* gene in barley is also related to leaf width and plant height. The homologous genes in rice were *NAL1* and *PLANT ARCHITECTURE AND YIELD1* (*PAY1*). The *NAL1* gene encodes an unknown protein related to polar auxin transport. A loss-of-function mutation in the *NAL1* gene leads to a narrow-leafed and dwarf phenotype (Qi et al., 2008). *PAY1* improves plant architecture by regulating polar auxin transport and altering endogenous indole-3-acetic acid distribution (Zhao et al., 2015). Consequently, the mechanisms by which *HvHNT1* influences leaf width and plant height could be similar to those associated with the *NAL1* gene. In this study, the narrower leaves of the *hnt1* mutant resulted from a significant reduction in the number of vascular veins per leaf (Fig. 3A), indicating that *hnt1* is related to vascular formation. In situ PCR analysis of the *HvHNT1* gene in leaves and stems also shows that the gene is preferentially expressed in vascular tissues (Fig. 7, C, D, F, and G). Therefore, *HvHNT1* may be involved in vascular development. During leaf differentiation, veins develop from leaf primordia in a specific pattern. The commonly accepted molecular model for vein development is the canalization-of-auxin-flow hypothesis, where *MONOPTEROS (MP)*, *PIN-FORMED1* (*PIN1*), and auxin are involved in the formation of leaf veins (Friml et al., 2003; Scarpella et al., 2006; Wenzel et al., 2007). *NAL1* affects vascular development as well as polar auxin transport in rice (Qi et al., 2008). Our preliminary results demonstrated that expression of the *PIN1* homologous gene was not impacted in the *hnt1* mutant, which differs from the rice *nal1* mutant (Qi et al., 2008). Furthermore, RNA interference of the *HvHNT1* gene increased tiller number but did not affect leaf shape in this study (Fig. 5E). Thus, the function of *HvHNT1* in barley may be similar to, but not the same as, *NAL1* in rice.

In Gramineae species, such as rice, wheat, and barley, tillers develop from the axillary meristem. Axillary bud development consists of two stages: (1) axillary bud formation when the bud is dormant, and (2) axillary bud outgrowth when bud dormancy is broken (Schmitz and Theres, 2005; Kebrom et al., 2012). Therefore, tillering depends not only on the initiation of axillary buds but also on the regulation of bud outgrowth. In this study, the *hnt1* mutant had almost four times more tillers than the wild type (Fig. 1B). The histological study showed no difference between the *hnt1* mutant and the wild type regarding the initiation of axillary buds, with one axillary bud in each leaf axil. However, bud outgrowth differed dramatically between the *hnt1* mutant and the wild type, with *hnt1* plants displaying significantly faster bud outgrowth than wild-type plants (Fig. 2). In other words, the *hnt1* mutant has a shorter dormancy duration of axillary buds than the wild type. In general, bud outgrowth is inhibited by the primary shoot apex, referred to as apical dominance. Therefore, we assume that more tillers per plant in the *hnt1* mutant is attributed to weakened or even lost apical dominance.

Our results indicate that *hnt1* is a pleiotropic mutant. Compared with the wild type, the *hnt1* mutant exhibited an abnormal phenotype. Both genetic analysis and transgenic validation demonstrated that all abnormal phenotypes were caused by a mutation in the *HvHNT1* gene, suggesting that *HvHNT1* is a crucial gene for tiller development regulation.

The results of the 2D proteomic analysis showed an accumulation of HvPPIase protein in *hnt1* that was not accompanied by changes at the transcriptional level. Together with the results obtained from the bioinformatic analysis and BiFC assay, we conclude that HvPPIase might be the substrate of the HvHNT1 protein. The PPIase superfamily in eukaryotes includes cyclophilins, FK506-binding proteins, parvulins, and PP2A phosphatase activators (Jing et al., 2015). Several plant *CYCLOPHILIN* (*CYP*)-like genes have been functionally characterized and are involved in a variety of physiological and developmental processes, including flowering, phytohormone signaling, and stress responses, and play important roles in regulating auxin signaling in plants (Trupkin et al., 2012; Ma et al., 2013; Yoon et al., 2016). Rice plants overexpressing *OsCYP19-4* showed cold-resistance phenotypes with significantly more tillers and spikes per plant (Yoon et al., 2016). The increase in tiller number in *OsCYP19-4*-overexpressing plants is consistent with the *hnt1* mutant phenotype. Mutations in the tomato cyclophilin *DGT* gene causes pleiotropic phenotypes, including a slow gravitropic response, reduced apical dominance, and altered vascular development (Balbi and Lomax, 2003; Lavy et al., 2012), and results in a similar phenotype to the *hnt1* mutant. Interestingly, the *dgt* mutant does not exhibit altered levels of auxin (Fujino et al., 1988) and appears to be affected in the expression of a subset of auxin-regulated genes in a tissue- and developmental stage-specific manner (Nebenführ et al., 2000; Balbi and Lomax, 2003), which is similar to that found in the *hnt1* mutant. There was no significant difference in endogenous auxin (indole-3-acetic acid) between *hnt1* and the wild type, but the transcriptional levels of many auxin-regulated genes, including some members of the *HvIAA* and *HvARF* families, changed in the mutant (Supplemental Fig. S3). It is well documented that auxin controls many aspects of plant physiology and morphology, including cell division, vascular tissue formation, adventitious root initiation, apical dominance, and tiller development. Therefore, more tillers per plant and narrower leaves in the *hnt1* mutant may be attributed to accumulation of the HvPPIase protein and changes in the expression of auxin-related genes (Supplemental Fig. S3). However, it is worth noting that the results of the BiFC assay on the physical interaction between HvHNT1 and HvPPIase have not been validated by an independent assay and that HvPPIase digestion by HvHNT1 has not been tested in vitro. Hence, further research is required to confirm if HvPPIase is a direct substrate of the HvHNT1 trypsin protein.

The identification of molecular players in plant development is important for genetic studies and plant breeding. In this case, the *HvHNT1* gene could be useful in barley breeding, as it affects tiller development, plant height, and leaf size. Moreover, this report describes the involvement of trypsin in controlling tiller development, plant height, and leaf size in a cereal crop.

## MATERIALS AND METHODS

### Plant Material and Tiller Observation

The *hnt1* mutant was isolated from the wild-type barley (*Hordeum vulgare*) ‘Vlamingh’ treated with γ-rays as follows. M1 seeds were sown in a field to select the mutant with more tillers per plant in M2 and then was progressed to M5 via self-pollination. The M5 seedlings were genetically and phenotypically stable and named the *hnt1* mutant. In addition, a DH population consisting of 269 lines derived from a cross between *hnt1* and cv Boluke was developed for use in this study. All DH lines and the two parents were grown in an experimental field at the Department of Agriculture and Food in Western Australia. Tillers per plant and leaf width were recorded at the heading stage, and plant height and 1,000 kernel weight were measured at maturity. Both *hnt1* and the wild-type cv Vlamingh were grown in a growth chamber with a 14-h/10-h day/night cycle at 23°C/18°C. The dynamic change in tillers per plant was recorded weekly by counting tillers on each of 12 *hnt1* and wild-type plants.

### Morphological Analysis

The shoot apices in 15-d-old seedlings of wild-type cv Vlamingh and the *hnt1* mutant were obtained for anatomical observation with a stereomicroscope. A minimum of eight shoot apices were dissected for each genotype for analysis by scanning electron microscopy.

The rolled and mature leaves from wild-type and *hnt1* seedlings were embedded in 5% (w/v) agarose and sliced into 80-μm sections for leaf vein, cell number, and size counting using a microscope with a camera. Cell size was calculated using ImageJ software with 20 biological repetitions.

### Mapping of *HvHNT1*

The genomic DNA of all DH lines and the two parents was extracted using the cetyl-trimethyl-ammonium bromide method. In total, 360 molecular markers scattered across all chromosomes were selected to examine the parents for polymorphism. The genotypes of all lines were then determined using 114 polymorphic markers. Tillers per plant and leaf width of each DH line and the two parents were recorded at the heading stage. Because multitiller plants are consistently associated with narrow leaves, the phenotype of the DH population was scored as A for wild type and B for *hnt1* type. In this study, we used the trait score as a marker. Using polymorphic markers, a genetic linkage map was constructed using Joinmap 4.0 software (https://www.kyazma.nl/index.php/JoinMap/). The molecular markers located on both sides of the trait marker were regarded as primary segregation markers. The region between the primary segregation markers was considered the primary candidate region, where 24 primary recombination lines were identified.

New molecular markers, including simple sequence repeats and small insertions and deletions, were developed based on sequence differences between cv Morex and cv Barke. The physical map and genomic sequence data were obtained from the MIPS barley genome database (http://mips.helmholtz-muenchen.de/plant/barley). The new markers and recombination lines were simultaneously used to narrow the candidate region. High-confidence genes in the candidate region were confirmed based on Mayer et al. (2012). The identified polymorphisms were used to develop new markers (CAPS_F/R). The sequence alignment and primer design were performed using GENEIOUS software (https://www.geneious.com/).

### Genetic Complementation Analysis

A 6.75-kb genomic DNA fragment containing a promoter (3,078-bp upstream sequence), coding region (3,348 bp), and 3′ untranslated region (324-bp downstream sequence) of the *HvHNT1* gene was amplified from wild-type genomic DNA using primers HNT1-Pro*Hin*dIII-F and HNT1-ORF-Sac-R (Supplemental Table S1). The fragment was cloned into the *Hin*dIII and *Sac*I sites of the binary vector pCAMBIA1300 without the 35S promoter to create the transformation plasmid for the complementation test. The constructed complementation vector was introduced into *Agrobacterium tumefaciens* strain AGL1 using electroporation and transferred into the *hnt1* mutant using the callus infection method (Harwood, 2014). Based on the sequence information of the constructed genetic complementation vector, a PCR-based marker was designed to verify the transformed lines. The forward primer (RE_F) was designed based on the initial vector fragment, and the reverse primer (RE_R) was designed based on the 6.75-kb genomic DNA. The CAPS marker was also used to verify the transformed lines. In addition, RT-qPCR was conducted to determine the *HvHNT1* gene expression level in the five independent transgenic lines (T2), the wild type, and the mutant. Total RNA from leaf tissue of transgenic lines (T2), wild-type, and mutant seedlings at the tillering stage was extracted using a centrifugal filter kit according to the user manual (Takara). After RT, qPCR was performed using gene-specific primers Q-HNT1_F and Q-HNT1_R. The relative expression level of each transcript was obtained by normalization to the *HvACTIN* gene.

### Generation and Analysis of the *HvHNT1* Knockdown Lines

To generate the hairpin RNAi construct, we cloned a 300-bp fragment (37–263 bp from ATG) of *HvHNT1* cDNA as inverted repeats into the pANDA vector (Miki and Shimamoto, 2004) under the control of the maize (*Zea mays*) ubiquitin1 promoter and subsequently transformed the vector into *A. tumefaciens* strain AGL1. The primer (RNAi-clon_F/R) sequences used for amplifying a 300-bp fragment of *HvHNT1* cDNA are shown in Supplemental Table S1. Immature embryos of barley 'Golden Promise' were used for *A. tumefaciens*-mediated transformation. The positive transgenic lines were confirmed by primers RNAi_F and RNAi-clon_R (Supplemental Table S1). The expression levels of *HvHNT1* were determined in the leaves of the T2 generation RNAi lines and cv Golden Promise (wild type) using RT-qPCR with primers Q-HNT1_F and Q-HNT1_R and *HvACTIN* gene normalization. The phenotype, including tiller number, leaf width, and plant height, was calculated at the heading stage.

### Phylogenetic Analysis

Homologous protein sequences of HNT1 were obtained from the Arabidopsis (*Arabidopsis thaliana*), tomato (*Solanum lycopersicum*), maize, sorghum (*Sorghum bicolor*), wheat (*Triticum aestivum*), *Brachypodium distachyon*, and rice (*Oryza sativa*) genome databases using an HvHNT1 amino acid sequence with ViroBLAST (Deng et al., 2007). A neighbor-joining tree was built using amino acid sequences of predicted proteins using GENEIOUS via the neighbor-joining method.

### Gene Expression Analysis (RNA Isolation and RT-qPCR Analysis)

Total RNAs were isolated from the basal stem, leaf, leaf sheath, root, stem, and spike of wild-type plants at different growth stages, including seedling, tillering, stem elongation, booting, and heading, using a centrifugal filter kit according to the manufacturer’s instructions (Takara). First-strand cDNA synthesis was performed with 1 μg of total RNA using the PrimeScipt RT reagent kit with gDNA Eraser (Takara), according to the user manual. cDNA samples were diluted 3-fold, with 1 μL used for further analysis. qPCR analyses were performed using gene-specific primers Q-HNT1-F and Q-HNT1-R (Supplemental Table S1) in the reaction system of SYBR Green Supermix (Bio-Rad) on a Roche 480 real-time PCR machine (Roche). The basal stem of *hnt1* and wild-type seedlings in the tillering stage was also used to analyze the gene expression of auxin-related genes. The barley *ACTIN* gene was used as an internal control. RT-qPCR was carried out with three biological and technical replications. The 2^−ΔΔCq^ relative quantification method was used to evaluate quantitative variation. All primers are listed in Supplemental Table S1.

### In Situ PCR

The in situ PCR analysis of *HvHNT1* and *HvPPIase* was performed using a described method (Athman et al., 2014) with several modifications. Barley leaf and basal stem samples from the seedling and booting stages were immersed in ice-cold FAA (containing 63% [v/v] ethanol, 5% [v/v] acetic acid, and 2% [v/v] formaldehyde) for 3 h. The samples were then embedded in 5% (w/v) agarose and sectioned to 80 μm. Ten sections were collected in one tube to perform the DNase treatment, and RT-qPCR was performed using the primers Q-HNT1-F2/R2 and Q-PPIase-F2/R2 listed in Supplemental Table S1. Sections were then treated by blocking the treatment, anti-DIG-AP binding, and staining with BM Purple AP substrate (Roche). After staining, the sections were washed and mounted in 40% (v/v) glycerol and observed with a Leica microscope. The negative controls followed the preparation of the test samples, but with the reverse transcriptase enzyme omitted. The positive control was carried out with the ribosomal 18S transcript; the primers are listed in Supplemental Table S1.

### Proteomic Analysis

Total proteins from the base stem tissues of the wild type and *hnt1* mutant at the four-leaf stage were subjected to 2D electrophoretic proteomic analysis. Three biological and two technical replicates of 2D gel images were scanned using a GS-800 2DE scanner (Bio-Rad), and protein spots were analyzed with ImageMaster 2D (Amersham Biosciences). To compare spot quantities between gels, the spot volumes were normalized as a percentage volume of three biological replicates. The significantly differentially expressed protein spots were excised and digested by trypsin for LC-MS/MS analysis. The procedure was performed as described by Wu et al. (2014) with some small modifications.

### Subcellular Localization and BiFC in Barley Protoplasts

For the constructs transiently expressing in barley leaf protoplasts, the coding regions of *HvHNT1* were amplified and cloned into pCAMBIA1300-YFP and BiFC vectors pSAT1-cEYFP-C1-B, and the coding regions of *HvPPIase* were amplified (primers are listed in Supplemental Table S1) and cloned into pCAMBIA1300-RFP and BiFC vectors pSAT1-nEYFP-C1, using the homologous recombination technology (Vazyme ClonExpress II One Step Cloning kit) to generate HvHNT1-YFP, HvHNT1-cYFP, HvPPIase-RFP, and nYFP-HvPPIase, respectively.

Barley protoplast isolation and polyethylene glycol-mediated transformation were performed according to Bai et al. (2014) with some minor modifications. In brief, the primary leaves of barley cv Golden Promise were cut into small strips, followed by cellulase R10 and macerozyme R10 (Yakult Pharmaceutical) digestion, washing, and collection. Ten milligrams of plasmid DNA of each construct was transformed into 0.2 mL of protoplast suspension. After incubation at 25°C in the dark for 12 to 18 h, fluorescence signals in barley protoplasts were detected. Confocal microscopy images were taken using an LSM780 confocal laser scanning microscope (Zeiss).

### Supplemental Data

The following supplemental materials are available.

**Supplemental Figure S1.** *HvHNT1* expression in various tissues at different growth stages (supplemental information for Fig. 7).
**Supplemental Figure S2.** In situ PCR analysis of the *HvPPIase* gene.
**Supplemental Figure S3.** Gene expression levels of auxin-related genes between *hnt1* mutant and wild-type seedlings.
**Supplemental Table S1.** Information on the primers used in this study.
**Supplemental Table S2.** The 32 high-confidence genes in the mapping region.
